# Rubidium

**Published:** 1865-02

**Authors:** 


*Rubidium.*—[illegible] rare alkaline metal [illegible] M. Bunsen in certain mineral waters by the spectrum analysis has been recently found by Grandeau in the ashes of beet root, tobacco, coffee tea and grapes. The spectrum of tobacco gave bands characteristic of lime, lithium, potassium and rubidium. These facts show that rubidium is one of the most widely diffused bodies in nature, since vegetables of the most diverse kinds are found to take it up from the soil. This opens a field of research in vegetable chemistry into which Mr. Grandeau proposes to enter.

